# Laparoscopic Appendectomy Using the Surgical-Glove Port Through an Umbilical Incision: A Single-Center Retrospective Study

**DOI:** 10.7759/cureus.24512

**Published:** 2022-04-26

**Authors:** Tran Que Son, Tran Hieu Hoc, Vu Duc Long, Tran Thanh Tung, Nguyen Minh Tuan, Bui Minh Hue, Nguyen Van Minh, Nguyen Toan Thang

**Affiliations:** 1 Department of Surgery, Hanoi Medical University, Hanoi, VNM; 2 Department of General Surgery, Bach Mai Hospital, Hanoi, VNM; 3 Emergency Center (A9), Bach Mai Hospital, Hanoi, VNM; 4 Anesthesia and Resuscitation Center, Bach Mai Hospital, Hanoi, VNM

**Keywords:** sila (single-incision laparoscopic appendectomy), sils (single-incision laparoscopic surgery), surgical-glove port, single-port laparoscopy, appendectomy

## Abstract

Background

Single-incision laparoscopic appendectomy (SILA) has recently emerged as a promising alternative for the management of acute appendicitis. This study aimed to compare the surgical outcomes of the SILA with those of three laparoscopic appendectomies (TLA) procedures using the existing equipment, the 10-mm laparoscope, and the surgical-glove port method.

Methodology

Between February 2021 and February 2022, this single-center retrospective study examined 68 patients who underwent laparoscopic appendectomy by a single surgeon. The study excluded patients with severe appendicitis, grade IV-V, following the American Association for the Surgery of Trauma classification. Clinical outcomes were analyzed, including operation time, hospital stay, postoperative pain, and postoperative morbidity.

Results

There were no statistically significant differences between SILA and TLA patients, respectively, in operation time (37.5 minutes vs. 35 minutes, p = 0.261) and the median duration of hospitalization (three days vs. three days, p = 0.929). There was no difference in the mean visual analog scale score between the two groups on the first day (p = 0.852), second day (p = 0.540), and the day of discharge from the hospital (p = 0.686), as well as return to diet (two days vs. two days, p = 0.053). Two (10%) cases of short-term complications in the SILA group and one (2.1%) case in the TLA group were noted.

Conclusions

SILA performed through a handmade surgical-glove port is a safe and viable therapy option for mild-to-moderate appendicitis. When the hospital lacks a specialized laparoscopic single-incision surgical system, this technique should be used on patients.

## Introduction

Laparoscopic appendectomy (LA) is now widely accepted as the gold standard for acute appendicitis, even in the most complex cases [1]. When performing LA, the laparoscope is often entered into the abdominal cavity via a periumbilical incision, followed by two more laparoscopic equipment entered and placed triangularly [2]. With the development of minimally invasive surgery, the number of ports has been decreased to improve cosmetic outcomes [2,3].

The single-incision laparoscopic appendectomy (SILA) is a virtually “scarless” procedure because a single port is located in the umbilicus [3]. It results in reduced postoperative pain, minor discomfort, and fewer surgical scares [4-6]. A new surgical concept usually raises many questions regarding safety, usefulness, appropriateness, applicability, and cost [7-9]. The cost of novel surgical procedures is always a significant issue in most countries. The use of these devices in SILA may lead to an increase in healthcare expenses. Numerous authors have developed their own SILA devices [1,3,7,10-12].

Hayashi et al. first developed the surgical-glove port in Japan [11]. Many surgeons have used the surgical-glove device in endoscopic gastrointestinal surgery such as cholecystectomy, gastrectomy, and colectomy [3,13,14]. For this component, access is relatively simple and it can be used with standard laparoscopic surgery equipment.

We conducted this study using a handmade surgical-glove port for LA. At our hospital, these were the first SILA procedures ever performed. The initial outcomes and obstacles encountered when using this method will provide invaluable experiences for colleagues worldwide.

## Materials and methods

Patient population

We performed a retrospective study from February 2021 to February 2022. This study included 457 patients who presented to our emergency department with right lower abdominal pain and were diagnosed with acute appendicitis by either clinical manifestations or imaging studies. Abdominal computed tomography (CT) and ultrasound (US) were performed depending on the patient’s clinical presentation. Patients who opted for nonoperative treatment were excluded; thus, finally, 413 patients underwent either laparoscopic or open appendectomy.

Inclusion and exclusion criteria

All SILA and three-port laparoscopic appendectomy (TLA) procedures in this study were performed by a single surgeon with more than 10 years of hepatobiliary and gastrointestinal surgical experience to standardize the surgical procedures. The inclusion criteria (for both TLA and SILA) were severe uncomplicated appendicitis (grades I and III according to the American Association for the Surgery of Trauma (AAST) classification). The grading of lesions was based on intraoperative (purulent and pseudomembranous) and pathological findings (perforated, acutely beneficial appendix; gangrenous appendix; phlegmon or abscess; generalized peritonitis). Patients with complicated appendicitis, such as a phlegmon or abscess, generalized peritonitis with grade IV-V following the AAST classification [15,16], and those with severe comorbidities were excluded from the study.

**Table 1:** Grading for appendicitis following AAST classification. AAST: American Association for the Surgery of Trauma

Grade	AAST disease grade description	Appendicitis
I	Local disease confined to the organ, minimal abnormality	Acutely inflamed appendix, intact
II	Local disease confined to the organ, severe abnormality	Gangrenous appendix, intact
III	Local extension beyond the organ	Perforated appendix with local contamination
IV	Regional extension beyond the organ	Perforated appendix with periappendiceal phlegmon or abscess
V	Widespread extension beyond the organ	Perforated appendix with generalized peritonitis

Data collection

Data and outcomes were compared between patients who received SILA (the single-port group) and those who received TLA (the three-port group).

Reviewed patient factors included age, sex, and body mass index (BMI). The following outcome variables were retrospectively collected: operative time, length of hospital stay, patient pain according to the visual analog scale (VAS), return to diet, and perioperative complications. Postoperative pain at rest was recorded after surgery on the first, second, and day of discharge using a 1-10 VAS scale [2].

All patients were thoroughly informed about the procedure, risks, and advantages of SILA and TLA. All patients in our study provided written informed consent. The study was approved by the Bach Mai Hospital Human Subjects Protection Committee (Code BM-2015-103, number 785/QĐ - BM). The unique research identification number (UIN) is researchregistry7726, available at researchregistry.com. The private information of all enrolled patients was carefully protected, and the study was conducted following the guidelines of the Declaration of Helsinki. This study is reported following the Strengthening the Reporting of Cohort Studies in Surgery (STROCSS) 2019 standards [17].

Operative techniques

**Figure 1:**
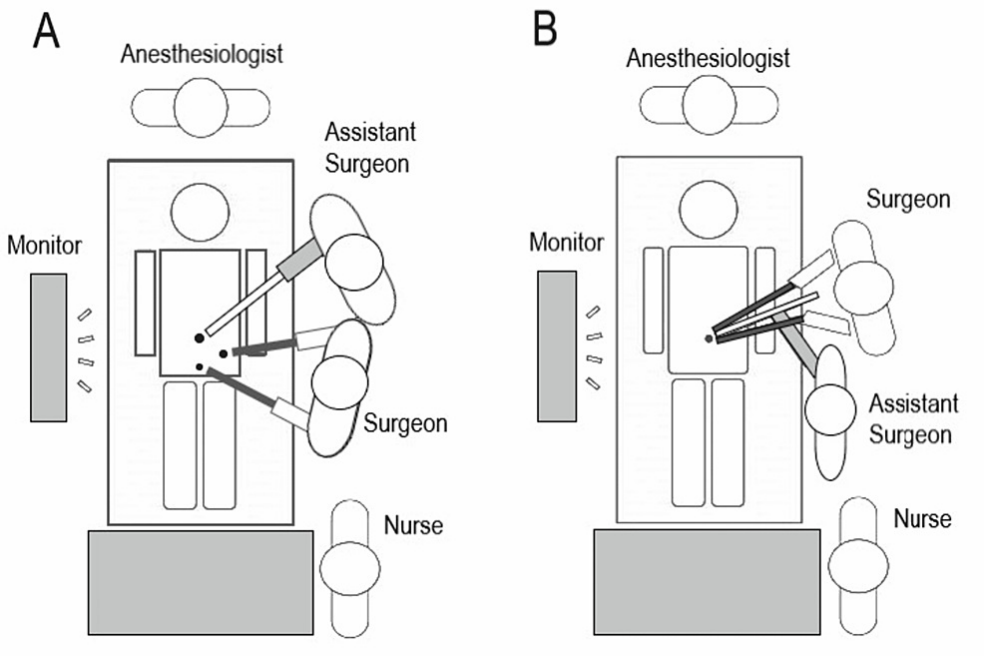
Positioning of the patient, surgeon, and first assistant surgeon.

All patients received a second-generation cephalosporin intravenously at induction of anesthesia. When performing SILA with the glove port, the umbilicus was cleaned thoroughly using cotton swabs. The patient was placed in a supine position with his left arm resting along his body (Figure 1).

**Figure 2:**
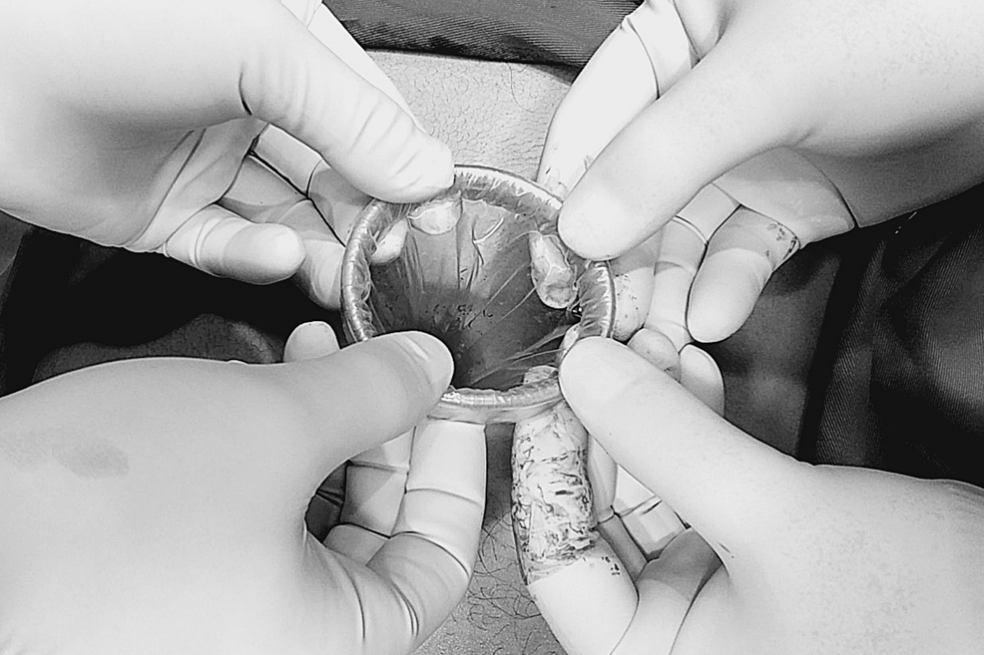
Placement of the wound protector.

A 2.5-3-cm long incision was made along the longitudinal direction of the umbilicus; subsequently, the fascia was divided to access the abdominal cavity. The finger was inserted into the wound to test the effectiveness of the fasciotomy [18]. An extra-small wound retractor (ALEXIS XS, Applied Medical, Rancho Santa Margarita, CA, USA) was placed, and the prepared surgical glove was fitted over the wound retractor (Figure 2).

**Figure 3:**
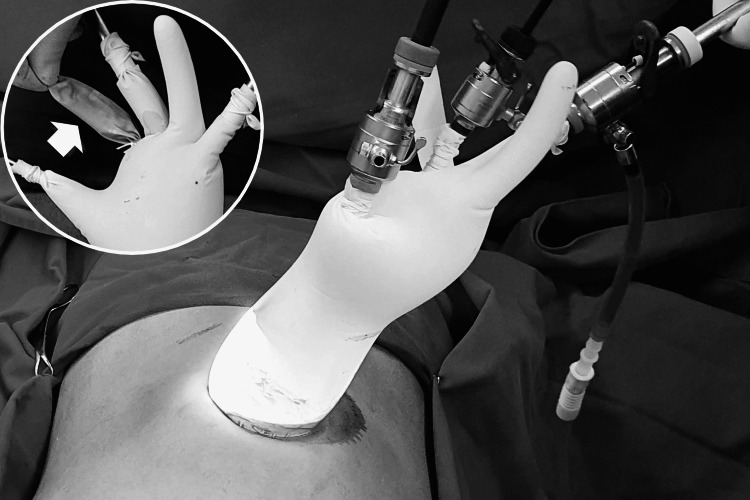
View from the exterior of the surgical-glove port: three ports were placed via the fingertips. A carbon dioxide insufflator attached to a 10-mm trocar was used to regulate pneumoperitoneum. The resected appendix was inserted into one of the digits following appendectomy (white arrow).

Three trocars, including two 10-mm trocars and one 5-mm trocar, were attached to the digits of a small-sized surgical glove before beginning the surgery (Figure 3).

After placing the single port into the abdominal wall, the patient was placed in the Trendelenburg position with the left side down. A 30-degree 10-mm laparoscope was used as the optical instrument. Laparoscopic appendectomy was performed conventionally. We only used unipolar cautery during SILA and TLA procedures.

There was no need for a vinyl bag to remove the resected appendix because the wound retractor protected the entire circumference of the incision from contamination.

**Figure 4:**
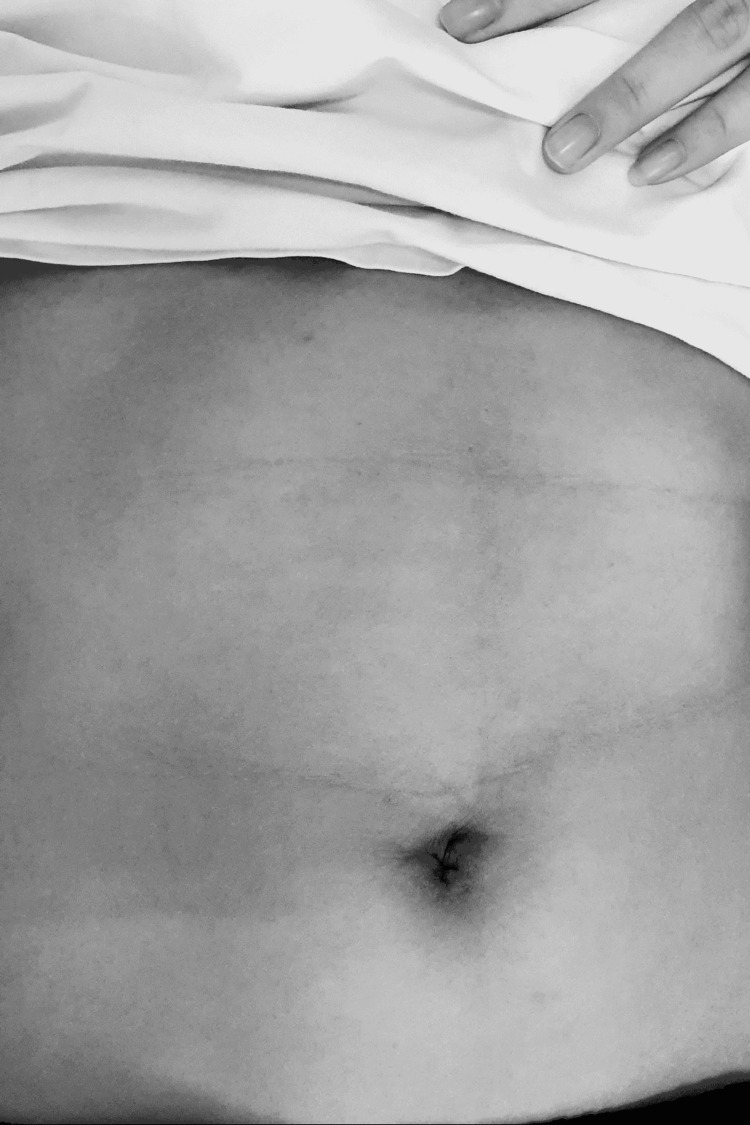
The umbilical scar after one month. The cosmetic effects of the transumbilical single-port laparoscopic appendectomy.

The appendix was extracted from the abdomen and placed into one of the glove’s free fingers. The digit was tied with silk to prevent spillage (Figure 2). Suturing was done with abdominal fascia closure with absorbable sutures (Safil 1.0, Braun, Spain) for both TLA and SILA surgery. For TLA, a single interrupted suture was used; for SILA, a single acronym suture was used. Top and bottom sutures were interrupted; one X-shaped suture ran through the middle of the abdominal fascia. The incision was sutured using Prolen 4.0 (Braun, Spain). After adequate closure, the immediate cosmetic effect was excellent (Figure 4).

When TLA was performed, a periumbilical incision was made for the initial approach of the laparoscope into the abdominal cavity. Under direct visualization, one more 5-mm and 10-mm trocars were inserted triangularly. Appendectomy was performed using the TLA method, and a vinyl bag was used for specimen retrieval. The fascia at the umbilical incision and the subcutaneous fat and skin at the other incisions were closed using absorbable sutures.

Follow-up

After surgery, intravenous antibiotics were continued throughout the hospital stay. Patient-controllable anesthesia was not used. To begin, pain management was managed by relaxation therapy utilizing breath control and muscle relaxation. Then, nonsteroidal anti-inflammatory drugs (NSAIDs) were administered by injection for pain that did not decrease. Patients were discharged when they were able to tolerate a regular diet.

Statistical analyses

Continuous variables are expressed as the mean ± standard deviation or median (interquartile range) and were evaluated using the Student’s t-test and Mann-Whitney U test. Categorical variables are expressed as the number (percentage) and were assessed using the chi-square test or Fisher’s exact test. All statistical analyses were performed using SPSS version 20.0 (SPSS Inc., Chicago, IL, USA). Results were considered statistically significant at p < 0.05 with a two-tailed test.

## Results

A total of 68 patients were reviewed. SILA was performed on 20 patients, and TLA was performed on 48 patients. Compared to the TLA group, the SILA group was younger, and men were more dominant than women. There was no difference in BMI, American Society of Anesthesiologists, or histological lesions of the appendix (Table 2).

**Table 2:** Patient characteristics.

Characteristic	Group 1 (n = 20)	Group 2 (n = 48)	P-value
Age (year)	21.5 (15–33.7)	33 (22.5–47.7)	0.019^a^
Gender			0.035^µ^
Male	15	22	
Female	5	26	
Body mass index (kg/m^2^)	21 (20.1–21.8)	20.8 (20.2–21.7)	0.549^a^
American Society of Anesthesiologists score			0.96^µ^
1	17	41	
2	2	4	
3	1	3	
Preoperative white blood cell count (/μL)	13.3 (12.1–14.5)	13.2 (11.9–14.3)	0.332^a^
Severity of appendicitis			1.000
Catarrhal	17	39	
Gangrenous appendix	3	9	

There was no difference in operation time between the SILA group (37.5 minutes) and the TLA group (35 minutes) (p = 0.261). The median hospital stay was three days in the SILA group and three days in the TLA group (p = 0.929). There was no difference in the mean VAS score between the two groups on the first day (p = 0.852), second day (p = 0.540), and the day of hospital discharge (p = 0.686). For all SILA patients, appendiceal stumps were closed with Hem-o-lok®. There were two cases of postoperative complications in the SILA group, including one patient with a wound infection. The remaining patient required reoperation two weeks after the initial operation due to an intra-abdominal abscess. There was no significant difference in the rates of complications between the two groups (p = 0.234) (Table 3).

**Table 3:** Postoperative clinical data. VAS: visual analog score

Variable	Group 1 (n = 20)	Group 2 (n = 48)	P-value
Operative time (minutes)	37.5 (32–45)	35 (30.25–40)	0.261^a^
Postoperative hospital stay (days)	3 (3–4)	3 (3–4)	0.929^a^
Control of appendix artery, n (%)			0.264^µ^
Monopolar electrocautery	9 (45)	14 (29.2)	
Hem-o-lok®	11 (55)	34 (70.8)	
Control of appendix stump, n (%)			0.254^µ^
Purse-string suture	0	5 (10.4)	
Sliding Roeder knot	0	1 (2.08)	
Hem-o-lok®	20 (100)	42 (87.52)	
Conversion	1 (5.0)	0 (0.0)	0.294^µ^
Time to diet (days)	2 (1–2.75)	2 (2–3)	0.053^a^
Postoperative complications, n (%)			0.234^µ^
Wound infection	1 (5)	0 (0)	
Intra-abdominal abscess	1 (5)	1 (2.1)	
VAS day 1	5 (3.25–5.75) 4.55 ± 1.32	5 (3–5) 4.67 ± 1.5	0.944^a^ 0.852^b^
VAS day 2	3 (2–3.75) 3.15 ± 1.14	3 (3–4) 3.48 ± 1.22	0.240^a^ 0.540^b^
VAS discharge hospital	2 (1–2) 1.85 ± 0.74	2 (2–3) 2.15 ± 0.77	0.147^a^ 0.686^b^

**Figure 5:**
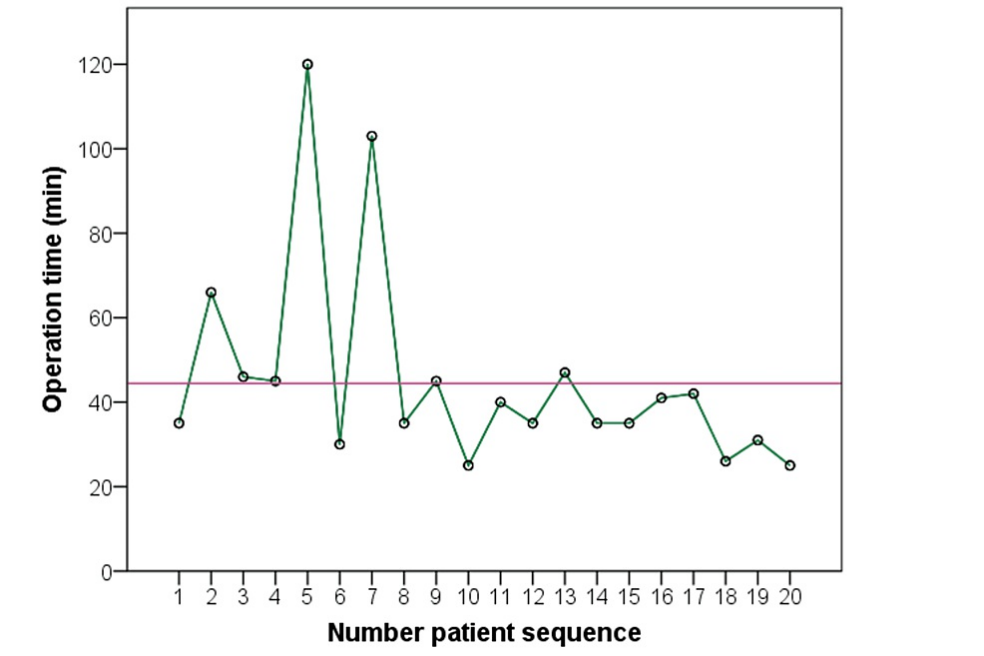
Reduced operation time in the transumbilical single-port laparoscopic appendectomy group based on surgical experience. The red line displays the average duration of the operation.

Reduced operation time in the transumbilical single-port laparoscopic appendectomy group was noted based on surgical experience (Figure 5).

## Discussion

In 2019, an estimated 17.7 million acute appendicitis cases were reported worldwide, with 228 cases per 100,000 people. Over 33,400 people died in the same year, with a death rate of 0.43 per 100,000 people [19]. Since its first description in 1983 by Semm [20], LA has been widely applied to treat complicated and uncomplicated appendicitis [4,6]. Compared to open surgery, LA has less postoperative pain and low doses of analgesics. It allows for early activities, food intake, and a short stay in the hospital. Thus, patients can return early to normal activities and have fewer cosmetic problems after surgery [4,21,22].

The incidence of acute appendicitis is in the 15-19-year age group [19], and cosmetic results are significant in this age group [23]. Pelosi and Pelosi first introduced single-incision laparoscopic surgery (SILS) in 1992 [24]. To date, SILS has been the subject of much criticism as a novel procedure, particularly for its complexity and cost; this attitude is comparable to what laparoscopic surgery received in the late 1980s [8]. However, SILS is widely applied in many operations because it has several advantages, such as cosmetic incisions reducing wound pain [1,3]. Furthermore, this technique may be converted to traditional surgery when necessary.

The overall complication rate of SILA ranges from 0.9% to 14.9% [6,18,25], with laparotomy conversion rates of 0% to 0.2% [4,21], and additional ports of 0.2% to 0.7% [4]. This shows the feasibility and safety of this surgical technique. The rate of surgical site infection of SILA has been reported to be 3% to 12.5% [4,6,10,25,26]. On the other hand, wound infection is more prevalent in the narrow (p = 0.027) and deep navel site (p = 0.384) [26]. First, patients with a small umbilicus are more prone to poor hygiene due to cleaning the area. Second, fascia excision often takes longer than skin excision. This may result in more significant injury to the subcutaneous tissue. Third, due to an improper suture of the dermis, particularly at the umbilicus. An extension of the umbilical incision may help prevent surgical site infections in patients with this umbilical morphology [26]. Many randomized controlled trials comparing SILA and TLA have shown similar surgical outcomes regarding operative time, length of hospital stay, and postoperative complications [4,5,8,12].

Nonetheless, SILA often demands a higher level of skills and expertise than TLA. Conflict of instruments used, absence of triangulation, and difficult retraction are the main technical issues in minimally invasive single-site surgery today [1,9,23]. Many generations of components, access, and retracting technologies have been designed to improve the method to overcome the above-mentioned obstacles [9].

However, the higher cost of SILA compared to the already expensive TLA remains a disadvantage that restricts the use of this method. One of the reasons for the higher cost of SILA compared with conventional laparoscopic surgery is a stapler or other devices for appendix stump closure [7,8]. In Japan, the end-loop costs 4,333 yen and a stapler (Echelon 60) costs 31,000 yen plus 32,000 yen for the cartridge (gold 60) [25]. A glove port and a standard three-port system (one 12-mm port and two 5-mm ports) cost 250 US dollars (USD) and 290 USD, respectively, according to the Korean Health Insurance [5]. Villalobos Mori et al. showed that SILA costs were significantly higher due to the Covidien SILS Port, which costs about twice as much as the combined cost of the three trocars used for TLA [8].

Consequently, more recently, SILS with a self-made glove port has been proposed to reduce surgical costs while maintaining aesthetics and effectiveness [2,3,7,10,11,23]. It is simple to use and adapts easily to the abdominal wall, even in obese patients. The two rings of the wound retractor can prevent subcutaneous emphysema, port-site infection, and bleeding. Due to the elasticity of surgical gloves, the equipment can efficiently perform a wide range of twisting and lifting actions without limiting their amplitude. The umbilical incision is minimized, reducing postoperative pain and the surgery site hernias [26]. Single-incision laparoscopic instruments are designed with three to four ports. Meanwhile, the glove port has five ports with no size limit [3]. Single-incision laparoscopy is a challenging procedure that requires extensive training. Even in developing nations like Vietnam, using a glove port will save the training expenses associated with the purchase of surgical equipment.

This approach has some disadvantages. First, because instrument conflicts frequently occur during surgery, cooperation between the camera holder and the surgeon is essential. Second, operating the instruments is not as stable as a standard three-trocar or single-port device due to the lack of a fixed point. Therefore, we placed the patient supine with the left arm along the body. The surgeon should take a position near the patient’s head, while the assistant should be below (Figure 1). Additionally, the surgeon must be skillful in instrument crossing movements during surgery.

Some surgeons invert the appendix stump using the purse-string suture during open appendectomy [18,25], with ENDOLOOP® simple ligation [23] and stump closure with invaginating suture [27] to avoid postoperative residual abscess. However, these patients had mild appendicitis according to the AAST classification [15]. Therefore, we adopted a Hem-o-lok® clip to ligate the artery and the appendix stump (Figure 4). Only two patients had an unusually long operational time (105 and 120 minutes). These are two cases with abnormal locations behind the cecum and under the liver, wherein one patient had complications that required reoperation due to a remaining intra-abdominal abscess (Table 3).

Many studies have suggested that SILA is technically more challenging for perforated appendicitis [2,6]. Complicated appendicitis makes dissection extremely difficult because of the significant intraperitoneal adhesion [26]. The authors attempted to resolve this problem by expanding the umbilical incision for the intracorporeal instruments conversion to multiport laparoscopic or open appendectomy [26].

The operation time ranges from 35 to 85.8 minutes depending on the study [1,3,7,10,28]. Our median operative time was 37.5 minutes, which was lower than other studies. Perhaps because the appendix was uncomplicated, the artery and appendix stump were occluded with Hem-o-lok® clips. Although the SILA median time was longer than the TLA time, no statistically significant difference was noted (p = 0.261), suggesting that this slight difference was caused by the extra time spent establishing the surgical-glove port and closing the incision [8].

Our study found no difference in VAS scores for SILA and TLA on the first day (4.55 vs. 4.67), the second day (3.15 vs. 3.48), or the day of hospital discharge (1.85 vs. 2.15). However, the difference in pain between SILA and TLA remains debatable. According to some studies, SILA is more painful than TLA, particularly during exertion and coughing [8,12]. Thus, numerous studies have revealed no difference in pain scores between the two procedures [1,5]. The incision length for SILS is 2.5-3 cm, while for TLA it is approximately 2.5 cm. This could be because the SILA incision is large, but the TLA incision is somewhat numerous. Similarly, SILA incises just the aponeurosis, unlike TLA which penetrates muscles, increasing the risk of vascular injury.

In our hospitals, introducing new operating procedures and materials into the operating room is more complex, with only essential laparoscopic equipment being accessible. Even with these difficulties, SILA was easily performed using the surgical-glove port and standard laparoscopic instruments. The wound retractor was the single additional operation material used in all cases.

There was no difference in surgical results between the two groups when performed by an experienced surgeon. However, the study sample excluded patients with severe complications of grade IV-V. Hence, our study has certain limitations. This was a retrospective study with a small sample size conducted in a single center. Two groups of patients were not randomized. There are certain selection biases in this study. We have not yet evaluated patients’ satisfaction with the incision’s cosmetics. We will continue to pay attention to this issue in the future.

## Conclusions

While the study excluded patients with severe appendicitis, SILA performed through a glove port is a safe and viable option for mild-to-moderate appendicitis. We anticipate that the surgical-glove port will become more widely used because of its steep learning curve. This technique should be used on children and adolescents when the hospital lacks a specialized laparoscopic single-incision surgical system.
